# A Novel Strategy for the Demulsification of Peanut Oil Body by Caproic Acid

**DOI:** 10.3390/foods12163029

**Published:** 2023-08-12

**Authors:** Yuhang Gao, Yanzhao Zheng, Fei Yao, Fusheng Chen

**Affiliations:** College of Food Science and Engineering, Henan University of Technology, No. 100 Lian Hua Rd., Zhengzhou 450001, China; gaoyuhang98@126.com (Y.G.); 18338187509@163.com (Y.Z.); yaofeixin@163.com (F.Y.)

**Keywords:** peanut oil body, caproic acid, demulsification, oil quality

## Abstract

The aqueous enzymatic method is a form of green oil extraction technology with limited industrial application, owing to the need for the demulsification of the oil body intermediate product. Existing demulsification methods have problems, including low demulsification rates and high costs, such that new methods are needed. The free fatty acids produced by lipid hydrolysis can affect the stability of peanut oil body (POB) at a certain concentration. After screening even-carbon fatty acids with carbon chain lengths below ten, caproic acid was selected for the demulsification of POB using response surface methodology and a Box–Behnken design. Under the optimal conditions (caproic acid concentration, 0.22%; solid-to-liquid ratio, 1:4.7 (*w*/*v*); time, 61 min; and temperature, 79 °C), a demulsification rate of 97.87% was achieved. Caproic acid not only adjusted the reaction system pH to cause the aggregation of the POB interfacial proteins, but also decreased the interfacial tension and viscoelasticity of the interfacial film with an increasing caproic acid concentration to realize POB demulsification. Compared to pressed oil and soxhlet-extracted oil, the acid value and peroxide value of the caproic acid demulsified oil were increased, while the unsaturated fatty acid content and oxidation induction time were decreased. However, the tocopherol and tocotrienol contents were higher than those of the soxhlet-extracted oil. This study provides a new method for the demulsification of POB.

## 1. Introduction

The peanut (*Arachis hypogaea* L.) is an important oilseed crop grown worldwide. The annual peanut output of China accounts for 36% of its global production. Peanuts are rich in oil, protein, and carbohydrates, and are commonly used for peanut oil extraction and consumption as food (such as peanut butter) [1,2]. Peanut oil contains about 80% unsaturated fatty acids and other nutrients including tocopherol, phytosterol, and squalene, resulting in an effective reduction in the incidence of diabetes and cardiovascular diseases, and a delayed progression of atherosclerosis [3,4].

Traditional peanut oil extraction methods include cold pressing (CP), solvent extraction (SE), and aqueous extraction (AE). The peanut oil extraction rate using CP is low, which limits its industrial application. The peanut oil extraction rate using SE is high, but its problems include solvent residue and production safety. The peanut meal obtained after high-temperature desolvation can only be used as feed, leading to wasted protein resources [5,6]. AE is applied to crushed oil plants using water as the extraction medium, wherein the oil and water are then separated by utilizing the different affinities of the different components in the materials for water, and differences in the oil and water densities. However, the extraction rate of AE is low, with most oil remaining in the oilseed cells in the form of oil bodies (OBs) [7]. Aqueous enzymatic extraction (AEE) is based on AE with added enzyme preparation to enzymatically hydrolyze the oilseed cell wall, resulting in an accelerated release of oil/OBs from the oilseed cells [8]. Commonly used enzyme preparations are divided into protease (Alcalase 2.4L, flavor protease, and papain) and glycosidase (cellulase, hemicellulase, pectinase, and viscozyme L) categories [9,10]. During protease use, storage proteins and oil body interface proteins (OBIPs) are enzymatically hydrolyzed into small peptide molecules, resulting in a low protein extraction rate and oil release from inside the OBs. However, glycosidase has no effect on these storage proteins and OBIPs, allowing for high-quality nonenzymatic protein and intact OBs to be obtained [11,12]. Owing to their good emulsification, thermal stability, and nutrient-rich properties, OBs are often used in natural emulsifiers, plant dairy products, and drug embedding, among other applications [13,14]. Accordingly, glycosidase is currently receiving greater interest.

Enzyme use is dependent on the oilseed type and cell wall composition. In previous work, our team found that viscozyme L efficiently degraded the cellulose, hemicellulose, and pectin in peanut cell walls, and accelerated the release of peanut oil bodies (POBs) and proteins, with highest yields of 93.67% and 76.84%, respectively [15,16]. When peanut oil is extracted via AEE (using glycosidase), the intermediate POBs must be demulsified to release the internal oil.

OBs are submicron-sized organelles that store triacylglycerols and are mainly composed of 94.21–98.17 wt% neutral lipids, 0.60–3.00 wt% OBIPs, and 0.60–2.00 wt% phospholipids [17]. These three components form a spherical structure with a neutral lipid core and interfacial membrane (protein–phospholipid interaction) outer layer [18]. OBs can remain stable under certain environmental conditions. Their stability is mainly determined by internal factors (the composition and structure of the interfacial membrane) and external environmental factors (pH and temperature). When OBIPs and phospholipids are hydrolyzed by protease or phospholipase, the structure and integrity of the OB interface membrane are destroyed and the internal oil is released [19,20]. The reaction system pH is close to the isoelectric point of OBIPs and OBs are demulsified due to the aggregation of the OBIPs [21]. Heating, freeze–thaw cycling, microwave irradiation, high-pressure CO_2_, inorganic salts, and enzymes can all cause OB demulsification [9,22,23,24]. Owing to their low demulsification rates, high costs, poor oil quality, and the subsequently required demulsification process, existing demulsification methods cannot meet the needs of industrial production, forcing researchers to seek new demulsification methods.

During the oil extraction process, oil is hydrolyzed to produce free fatty acids, and the hydroxyl hydrogen atom of the fatty acids can ionize to hydrogen cations. When these fatty acids reach a certain concentration, they can adjust the reaction system pH to demulsify OBs. Added fatty acids can be removed in the subsequent oil refining process. Odd-carbon fatty acids are mostly toxic, while fatty acids with more than ten carbon atoms are solid at room temperature. Therefore, even-carbon fatty acids with a carbon chain length below ten were selected for demulsification tests. In this study, POB was first extracted via AEE (viscozyme L), followed by the screening of fatty acids and the optimization of the demulsification process. The oil obtained by the fatty acid demulsification was compared with that obtained by pressing and Soxhlet extraction, and the demulsification mechanism was explored.

## 2. Materials and Methods

### 2.1. Materials

Ripe Yuhua-23 peanuts (20 to 30 capsules per tael, moisture content 5.0 wt%) were purchased at a local market (Zhengzhou, China). Pressed oil was gained using a piece of YKY-6YL-550 hydraulic equipment (Zhengzhou Bafang Machinery Equipment Co., Ltd. Zhengzhou, China) at 45 MPa for 30 min. Soxhlet-extracted oil (n-hexane extraction) was extracted using a Soxhlet extractor at 50 °C for 6 h. Viscozyme L (5086 U/mL, a complex plant hydrolase comprising the main ingredients cellulase, hemicellulose, and arabinase) was purchased from Novozymes (Bagsvaerd, Denmark). All the chemicals and reagents were purchased from Aladdin Reagent Co., Ltd. (Shanghai, China), and were of analytical reagent grade or higher.

### 2.2. Extraction of Peanut Oil Body by Aqueous Enzymatic Extraction

The POBs were extracted using AEE as described by Li et al. [25], with some modifications. The peanuts were mixed 1:5 (*w*/*v*) with deionized water (weight noted as w), soaked at 4 °C for 8 h, washed with deionized water 2–3 times, and then re-added to the deionized water to weight w. The soaked peanuts were then crushed with a tissue shredder (C022E, Joyoung Co., Ltd., Shandong, China) for 2 min to form peanut milk. Viscozyme L (2.00%, *v*/*w*) was added for enzymatic hydrolysis at 50 °C for 2 h. The enzyme was inactivated at 95 °C for 2 min and then cooled to room temperature. The peanut milk was centrifuged at 5000 rpm for 10 min (DZ267-32C6, Anting Scientific Instrument Factory, Shanghai, China), the upper POBs were extracted, and the centrifugation was repeated 3 times to collect all the oil bodies. The oil body was stored at 4 °C and analyzed within 24 h.

### 2.3. Screening of Fatty Acids

Using a solid-to-liquid ratio of 1:5 (*w*/*v*), a temperature of 60 °C, and a time of 60 min, the pH value of the POB was adjusted to the POB interfacial protein isoelectric point (pH 4.50) using formic acid, acetic acid, butyric acid, caproic acid, and caprylic acid, respectively, and the demulsification rates were recorded. The demulsification rate was calculated using Equation (1):

(1) $$Demulsification\;rate\;(\%)=\frac{Weight\;of\;free\;peanut\;oil\;(g)}{Oil\;weight\;in\;peanut\;oil\;body\;\left(g\right)}\times 100$$

### 2.4. Optimization of Fatty Acid Demulsification

The fatty acid selected from the previous experiment was used to optimize the demulsification of the POBs. The optimal extraction concentration (%), solid-to-liquid ratio (*w*/*v*), time (min), and temperature (°C) were determined by a Box–Behnken design (BBD) using response surface methodology (RSM). The generalized polynomial model used to predict the corresponding variable is shown in Equation (2):

(2) $$Y=\beta_{0}+\sum \beta_{i}X_{j}+\sum \beta_{ij}X_{i}X_{j}+\sum \beta_{ii}X_{i}^{2}$$

where Y is the predicted response, β_0_, β_i_, β_ij_, and β_ii_ are the regression coefficients for intercept, linear, interaction, and square, respectively, and X_i_ and X_j_ are the independent coded variables. The coding levels of the response surface factors are shown in Table 1. Design expert software Version 8 (Stat-Ease, Inc., Minneapolis, MN, USA) was used to perform the data analysis and RSM.

### 2.5. Sodium Dodecyl Sulfate—Polyacrylamide Gel Electrophoresis (SDS-PAGE)

SDS-PAGE was used to analyze the composition of the OBIPs, as described by Zhou et al. [26]. The POB protein content was measured via Kjeldahl nitrogen determination, with 1:1.5 (*w*/*w*) sodium lauryl sulfate added to the elute OBIPs and the lower aqueous phase (protein phase) being extracted by centrifugation and mixed with the loading buffer in a ratio of 1:1 (*v*/*v*), to achieve a final protein content of 5 mg/mL. The concentrations of the concentrated gel and separating gel were 5% and 12%, respectively.

### 2.6. Physicochemical Properties

The acid value and peroxide value were determined using the AOCS official methods Cd 3d-63 and Cd 8-53 [27].

### 2.7. Fatty Acid Composition

The Folch’s method was used to extract lipids from the POB [28]. The oil was converted into fatty acid methyl esters according to the method described by Fozo et al. [29]. The fatty acid composition was determined using gas chromatography (Agilent Technologies chromatograph model 5975 inert XL Net Work GC system) equipped with a flame ionization detector. The gas chromatography conditions were as follows: HP-88 capillary column (100 m × 250 μm × 0.20 μm); programmed heating, increased from 140 °C (held for 1 min) to 240 °C (held for 20 min) at 4 °C/min; inlet temperature, 260 °C; and flame ionization detector temperature, 260 °C. Hydrogen (H_2_) was supplied by a hydrogen generator (SGH-300, Beijing Oriental Essence, Beijing, China) and flowed at a rate of 30 mL/min. Nitrogen (N_2_) was used as the carrier gas at a flow rate of 1 mL/min with a split ratio equal to 1:50, and air was generated by an air generator (QY-3, Qingchuan, Jinan, China) at a rate of 400 mL/min. The composition of the fatty acids was expressed as a percentage of the peak area.

### 2.8. Determination of Tocol (Tocopherol and Tocotrienol) Contents

A total of 0.5 g of peanut oil was thoroughly mixed in n-hexane (HPLC grade) and the volume was fixed into a 10 mL volumetric flask. The mixture was filtered through a 0.45 µm organic membrane and injected into a liquid phase vial for an HPLC analysis. The HPLC conditions were consistent with the method of Ji et al. [30]. Qualitative and quantitative determination was then performed according to the standard solution and standard curve.

### 2.9. Oxidation Stability

The oxidative stability was determined by measuring the oxidation time using Rancimat apparatus (Metrohm CH series743, Zofingen, Switzerland). At 120 ± 0.2 °C, the peanut oil (5.0 g) was charged with air at a rate of 20 L/h and the volatile compounds produced by the peanut oil were introduced into pure water. The resulting changes in the electrical conductivity of the pure water were then continuously observed.

### 2.10. Statistical Analysis

All the tests were performed in triplicate and the data were expressed as means ± standard deviation. Plots were drawn using Origin 8.5 software. The data were subjected to statistical analysis using the SPSS 17.0 software package (SPSS Inc., Chicago, IL, USA). One-way analysis of variance (ANOVA) was used to analyze the significant differences (*p* < 0.05), and significant differences (*p* < 0.05) within same index are represented by different lowercase letters (a–e).

## 3. Results and Discussion

### 3.1. Fatty acid Screening

**Figure 1:**
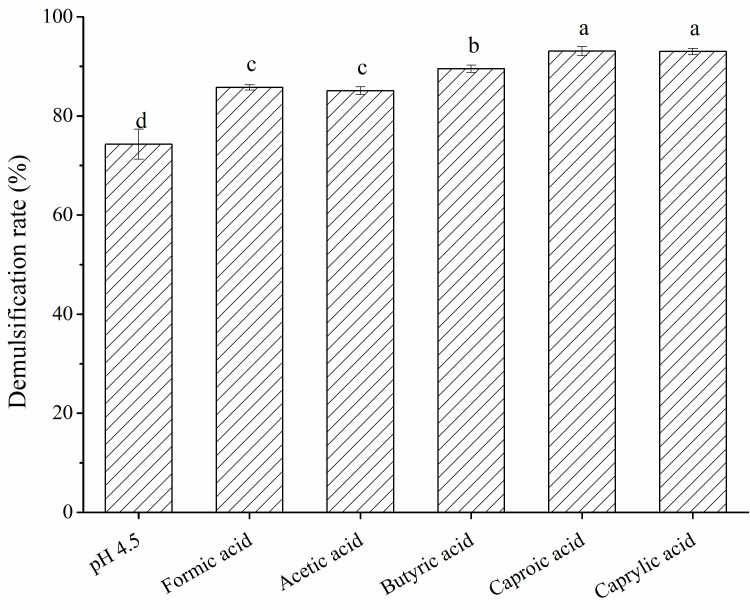
Effects of different fatty acids on the demulsification rate of peanut oil bodies. Different letters indicate significant differences among samples (*p* < 0.05).

Common short-chain fatty acids, including formic acid, acetic acid, butyric acid, caproic acid, and caprylic acid, were screened by addition to the POBs to obtain a POB pH value of 4.5 (the isoelectric point of OBIPs). The resulting demulsification rates are shown in Figure 1. Without a fatty acid addition, the POB demulsification rate at pH 4.5 was 74.29%, which was lower than that reported by Chabrand et al. (83.00%) [31]. This difference might be due to the different oilseed types, extraction methods, and OB compositions. The demulsification rate was significantly increased by the addition of different fatty acids, with caproic acid and caprylic acid affording demulsification rates of 93.10% and 93.01%, respectively. The improved demulsification rate with the fatty acid addition indicated that fatty acids effectively reduced the stability of the POB interface film, causing the release of the internal oil. As the most effective fatty acid, caproic acid was selected for the subsequent experiments.

### 3.2. Single-Factor Experiments

#### 3.2.1. Caproic Acid Concentration

**Figure 2:**
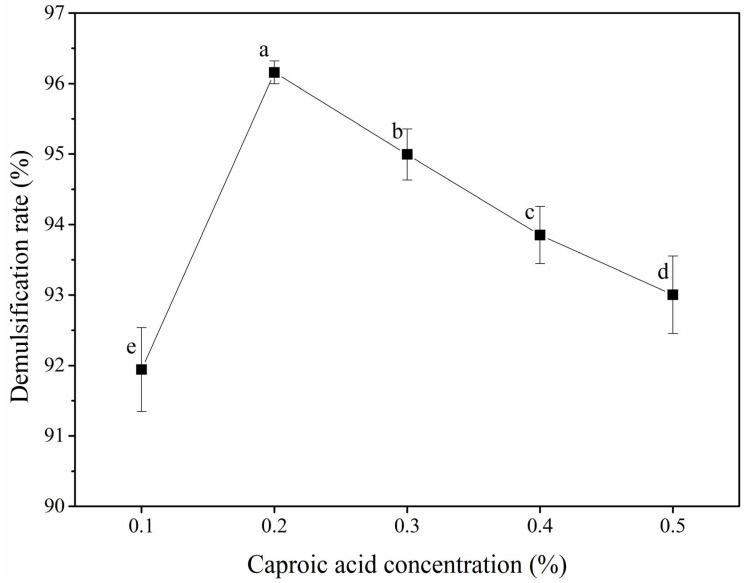
Effect of caproic acid concentration on the demulsification rate of peanut oil bodies. Different letters indicate significant differences among samples (*p* < 0.05).

The effect of the caproic acid concentration on the POB demulsification rate is shown in Figure 2. With an increasing caproic acid concentration, the POB demulsification rate showed an initial increase and subsequent decrease. When the caproic acid concentration was 0.2% (*v*/*w*), the demulsification rate was highest, at 96.16%. The POB interfacial membrane is mainly composed of interfacial proteins and phospholipids. As amphiphilic molecules, the solubility of the OBIPs was reduced at an acidic pH, and the interfacial adsorption and diffusion were inhibited, resulting in a decreased POB stability. The POB stability was worst at pH values close to the isoelectric point [32]. As a low-molecular-weight fatty acid, caproic acid can provide a large amount of hydrogen ions to adjust the reaction system pH, decreasing the pH value with an increasing caproic acid concentration.

When the caproic acid concentration was less than 0.20%, the demulsification rate increased with an increasing caproic acid concentration, while the reaction system pH decreased, albeit remaining higher than the isoelectric point of the OBIPs, such that the contact between the caproic acid and POBs was insufficient [33]. When the concentration was 0.20%, the reaction system pH was 4.53, which was close to the isoelectric point of the OBIPs, and the demulsification rate obtained was highest. However, with an increasing concentration greater than 0.20%, the pH value decreased and was far from the isoelectric point, and the POB demulsification rate decreased. Therefore, caproic acid concentrations of 0.10–0.30% (*v*/*w*) were selected for the response surface tests.

#### 3.2.2. Solid-to-Liquid Ratio

**Figure 3:**
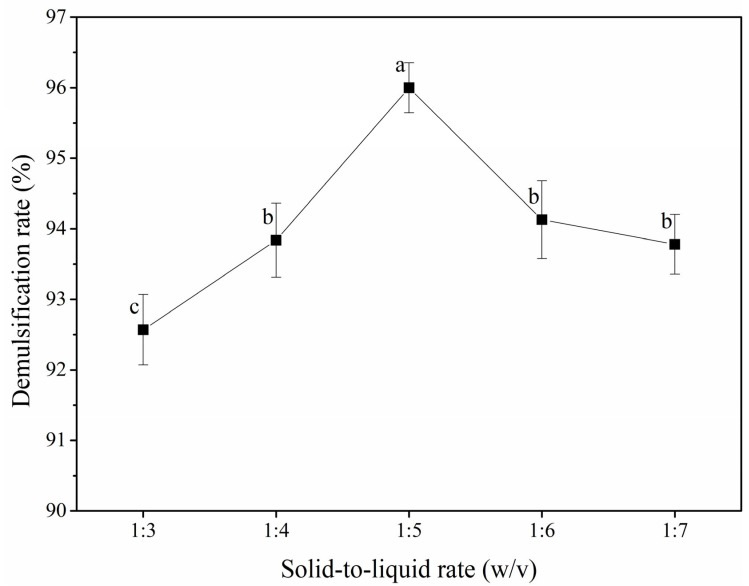
Effect of solid-to-liquid ratio on the demulsification rate of peanut oil bodies. Different letters indicate significant differences among samples (*p* < 0.05).

Figure 3 shows the effect of the solid-to-liquid ratio (ratio of POBs to water; 1:3–1:7, *w*/*v*) on the demulsification rate. With an increasing materials-to-liquid ratio, the POB demulsification rate initially increased and then decreased, with the maximum demulsification rate of 96.00% being achieved at a ratio of 1:5. The solid-to-liquid ratio determined the POB concentration, caproic acid concentration, and uniformity of the reaction system during the demulsification process [34]. When the solid-to-liquid ratio was less than 1:5, the reaction system had a high viscosity, such that caproic acid could not achieve full contact with the POBs [35]. A lower solid-to-liquid ratio resulted in a lower reaction system pH (below the isoelectric point of the OBIPs (4.50)) and, therefore, a decreased POB demulsification rate. When the solid-to-liquid ratio was greater than 1:5, not only was the caproic acid concentration reduced and the contact area between the caproic acid and POBs increased, but the reaction system pH was also greater than the isoelectric point of the OBIPs, resulting in a lower demulsification rate [36]. Finally, solid-to-liquid ratios of 1:4–1:6 (*w*/*v*) were selected for the response surface tests.

#### 3.2.3. Reaction Time

**Figure 4:**
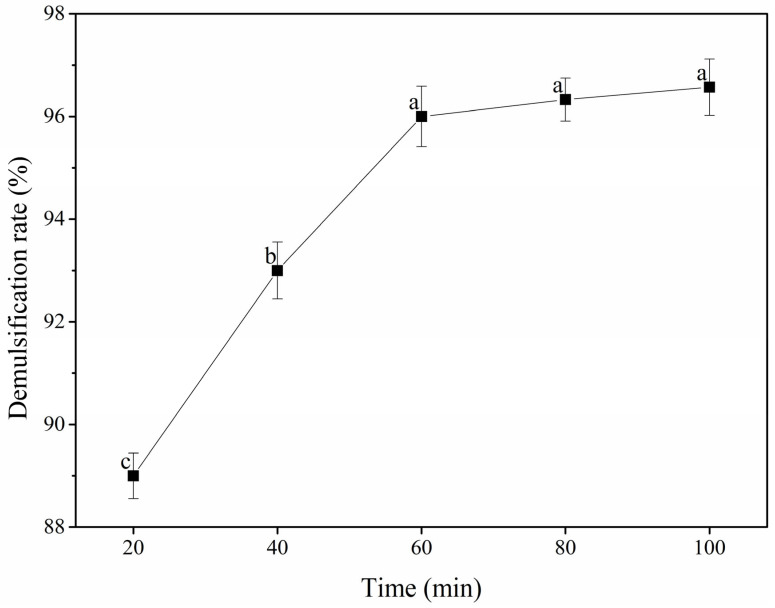
Effect of reaction time on the demulsification rate of peanut oil bodies. Different letters indicate significant differences among samples (*p* < 0.05).

The effect of the reaction time on the POB demulsification rate is shown in Figure 4. With an increasing reaction time in the range of 20–60 min, the POB demulsification rate increased significantly, reaching the maximum value of 96.00% at 60 min. As the reaction time was further increased, the demulsification rate showed a stable, gradual increase. The reaction time affected the POB demulsification rate through two mechanistic steps; initially, the caproic acid diffused into the POB surface and then entered the POB interior to promote peanut oil release [35]. Owing to its slightly water-soluble nature, the caproic acid diffused to the surface of the POBs slowly. Within 20–60 min, the reaction between the caproic acid and POBs became more extensive with an increasing time, resulting in an increased demulsification rate. An excessive reaction time might lead to the degradation of oil quality, unnecessary energy consumption, and by-product formation [37]. Therefore, reaction times of 40–80 min were selected for the response surface tests.

#### 3.2.4. Temperature

**Figure 5:**
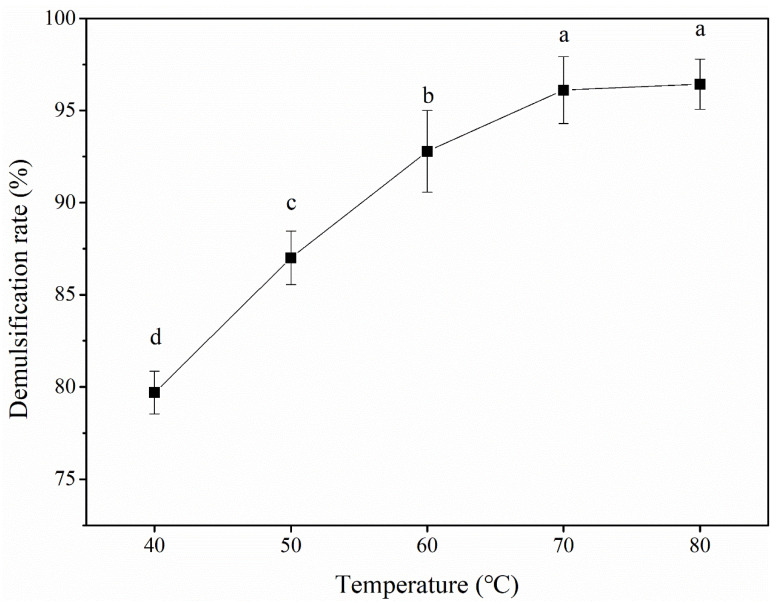
Effect of temperature on the demulsification rate of peanut oil bodies. Different letters indicate significant differences among samples (*p* < 0.05).

Figure 5 shows the effect of the temperature on the POB demulsification rate. The POB demulsification rate significantly increased with an increasing temperature between 40 and 70 °C. The highest demulsification rate of 96.11% was obtained at 70 °C. The demulsification rate plateaued at temperatures exceeding 70 °C. The increased reaction temperature reduced the surface tension and viscosity of the POBs, resulting in an improved diffusion of caproic acid in the reaction system and improved contact with the POBs. Furthermore, the temperature could cause the OBIP structure to expand, exposing more binding sites and increasing the demulsification rate [34]. When the temperature exceeded 70 °C, the POB demulsification rate did not change significantly, but the nutrients in the demulsified peanut oil that could not withstand high temperatures became denatured with an increasing temperature, resulting in decreased quality [37,38]. Finally, reaction temperatures of 60–80 °C were selected for the response surface tests.

### 3.3. RSM Model Development

With the demulsification rate as the response value (Y), and the caproic acid concentration (X_1_), solid-to-liquid ratio (X_2_), reaction time (X_3_), and temperature (X_4_) as independent variables, the corresponding experimental design and results are shown in Table 2. A total of 29 runs were performed in this experiment, with the demulsification rate ranging from 87.08% to 97.83%.

According to the results, regression fitting was conducted for each factor, and the following multiple regression equation was obtained, as shown in Equation (3):

Y = 96.71 + 1.45X_1_ + 0.28X_2_ + 1.68X_3_ + 2.26X_4_ + 0.60X_1_X_2_ − 0.65X_1_X_3_ − 0.43X_1_X_4_ −  0.64X_2_X_3_ − 0.31X_2_X_4_ − 0.65X_3_X_4_ − 2.70X_1_^2^ − 1.47X_2_^2^ − 1.82X_3_^2^ − 1.11X_4_^2^ (3)

The results of the variance analysis are shown in Table 3. The model *p* value was less than 0.0001, indicating that the model was significant, although the lack of fit was not significant (*p* > 0.05). The caproic acid concentration (X_1_), reaction time (X_3_), and temperature (X_4_) had extremely significant effects on the demulsification rate, while the solid-to-liquid ratio (X_2_) did not have a significant effect. The significance order of the factors was temperature > time > concentration. The correlation coefficient of the model (R^2^) was 0.9494 and the adjusted correlation coefficient (R^2^_adj_) was 0.8989. Therefore, the regression model predicted the effect of the caproic acid on the demulsification rate of the POBs.

### 3.4. Determination of Optimal Conditions

The optimal demulsification process predicted by the regression model had a caproic acid concentration of 0.22%, a solid-to-liquid ratio of 1:4.73 (*w*/*v*), a reaction time of 61.2 min, and a temperature of 78.9 °C; the predicted demulsification rate was 97.87%. To facilitate the actual operation, the predicted conditions were modified to a caproic acid concentration of 0.22%, solid-to-liquid ratio of 1:4.7 (*w*/*v*), reaction time of 61 min, and temperature of 79 °C. The actual demulsification rate was 97.64 ± 0.42%, representing a 0.23% difference from the predicted value, which indicated that the actual value was close to the predicted value, and the model was reliable and applicable for an accurate prediction of the POB demulsification rate.

### 3.5. Caproic Acid Demulsification Mechanism

**Figure 6:**
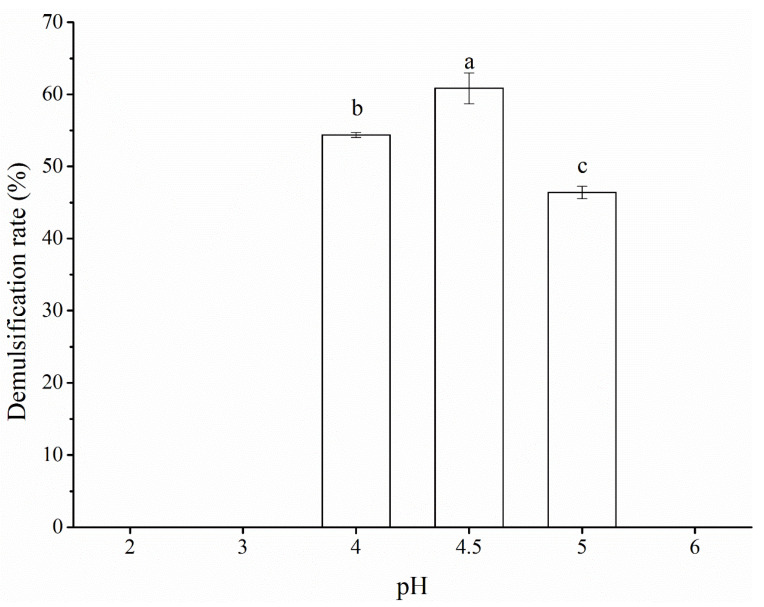
Demulsification rate of peanut oil bodies under different pH conditions. Different letters indicate significant differences among samples (*p* < 0.05).

As shown in Figure 6, under acidic pH conditions, the POBs were demulsified at pHs of 4.00, 4.50, and 5.00, with the maximum demulsification rate of 60.84% being obtained at a pH of 4.50. At a pH of 4.50, the OBIPs were at their isoelectric point and the proteins aggregated to release the oil inside the POBs. Owing to differences in the peanut raw materials and oil extraction methods, different POBs had different isoelectric points. The POBs extracted by Wang et al. [39] had an isoelectric point of pH 4.70, while those extracted by Tzen et al. [40] had isoelectric points of pH 5.00–6.00. The demulsification rate was lower than the 82.00% reported by Chabrand et al. [31], due to the demulsification rate not reaching the maximum value under the optimal conditions in this experiment.

**Figure 7:**
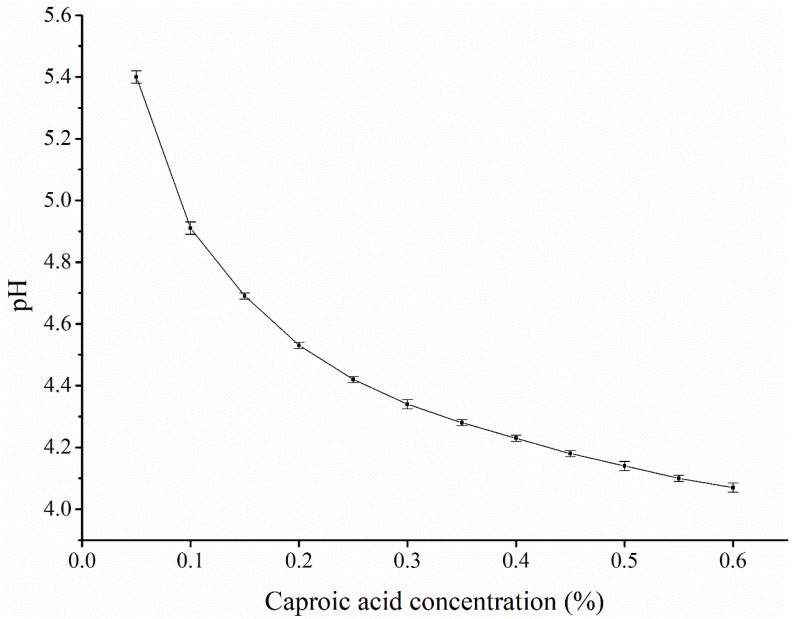
The pH of peanut oil bodies with different concentrations of caproic acid added.

**Figure 8:**
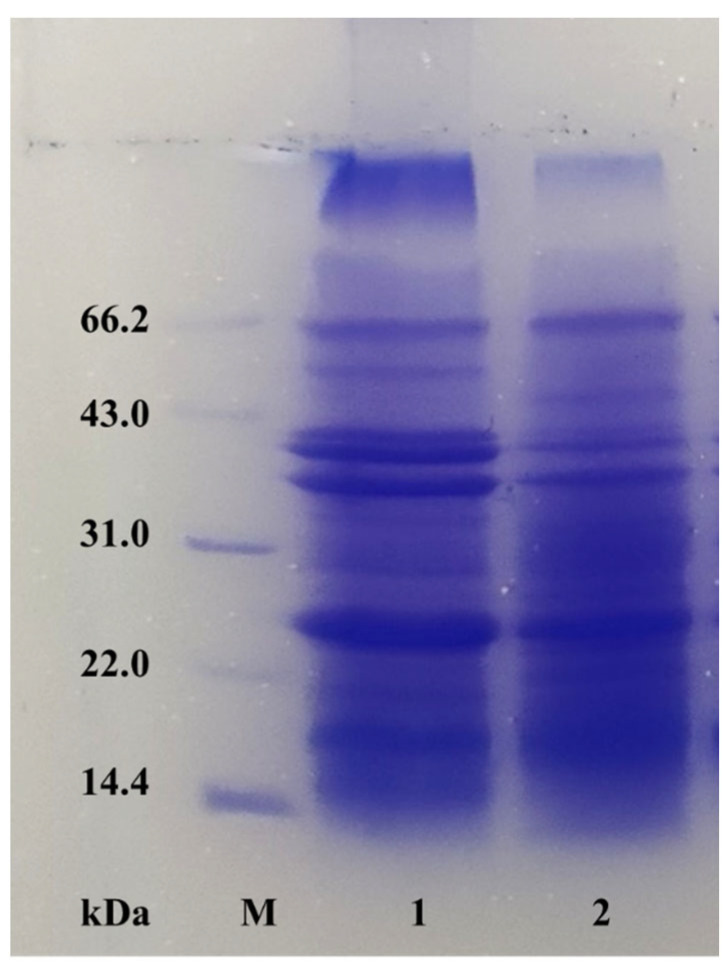
Interfacial protein compositions of peanut oil bodies before and after demulsification (M, band 1, and band 2 represent the marker, and peanut oil bodies before and after demulsification, respectively).

For the POBs and deionized water mixed at a 1:5 (*w*/*v*) ratio, the pH value of the reaction system after adding caproic acid at different concentrations is shown in Figure 7. With an increasing caproic acid concentration, the reaction system pH initially decreased sharply, and then decreased more slowly. Caproic acid was shown to release large amounts of hydrogen ions to adjust the pH value of the system. Compared with pH demulsification, caproic acid affords a higher demulsification rate. The addition of caproic acid could reduce the pH value of the reaction system, with the highest demulsification rate being obtained when the amount of caproic acid added caused the reaction system pH to reach the isoelectric point of the POBs. As shown in Figure 8, no difference in the composition of the OBIPs was observed before and after the demulsification with caproic acid, indicating that caproic acid adsorbed to the POB surface quicker and reduced the interfacial tension of the POBs more effectively, in comparison to the OBIPs [41]. In summary, the mechanism of caproic acid demulsification was divided into two parts. First, caproic acid reduced the reaction system pH, causing OBIP aggregation. Second, the caproic acid addition significantly reduced the interfacial tension and viscoelasticity of the interfacial film with an increasing caproic acid concentration, achieving demulsification [42].

### 3.6. Physicochemical Properties of Caproic Acid Demulsified Oil

As shown in Table 4, the acid value and peroxide value of the pressed oil were lowest among the three types of peanut crude oil, while the acid value and peroxide value of the caproic acid demulsified oil were significantly higher, at 0.62 mg KOH/g and 0.23 g/100g, respectively, than those of the pressed oil and soxhlet-extracted oil. The acid value of the caproic acid demulsified oil was the highest, perhaps due to a small amount of caproic acid being dissolved in the peanut oil during the demulsification process, and the high temperature of the caproic acid demulsification leading to oil hydrolysis, which increased the free fatty acid content. The peroxide value increased due to differences in the chemical composition of the lipids (free fatty acids, natural antioxidants, and pro-oxidants) and their tolerance to environmental factors, leading to oxidation reactions in the lipids [43,44]. The free fatty acids and peroxides in caproic acid demulsified oil can be removed by subsequent oil refining.

### 3.7. Fatty Acid Composition

The fatty acid composition of the crude peanut oil was analyzed using three different methods, as shown in Table 5. A total of nine fatty acids were detected. Among the saturated fatty acids, palmitic acid (C16:0) and stearic acid (C18:0) were the most abundant, with small amounts of behenic acid (C22:0) and lignoceric acid (C24:0) also being present. The oleic acid (C18:1) content was highest among the monounsaturated fatty acids, accounting for about 37.75–38.19% of the total fatty acid content. The only polyunsaturated fatty acid detected was linoleic acid (C18:2), accounting for 38.90–40.67% of the total fatty acid content. The different fatty acid compositions in the crude peanut oil were obtained using the three extraction methods. No erucic acid was detected in the pressed oil and soxhlet-extracted oil, while 0.25% of erucic acid was found in the caproic acid demulsified oil. The three crude oils had similar unsaturated fatty acid contents, at about 80%, with oleic acid and linoleic acid being the most abundant. Oleic acid is considered to be the healthiest dietary fatty acid, owing to its ability to lower cholesterol, regulate blood lipids, and lower blood sugar [45]. The oleic acid and linoleic acid contents were similar, with relative content ratios close to 1. The stability index, namely the oleic acid/linoleic acid (O/L) ratio, is usually used to evaluate the quality of peanuts and peanut products [46]. From the modern nutritional perspective, a high O/L value indicates a product with a better shelf life and higher nutritional value [47,48].

### 3.8. Total Tocopherol and Tocotrienol Contents

Tocopherols are important endogenous antioxidants that can inhibit the internal and external oxidation of vegetable oil, and improve the antioxidant capacity of vegetable oil by inhibiting primary oxidation in free radical chain reactions [49]. The degree of the inhibition of oil oxidation rancidity is positively correlated with tocopherol content [50]. Four types of tocopherols (α, β, γ, and δ) and two types of tocotrienols (α and γ) were detected in the three types of peanut oils, and their specific contents are shown in Table 6.

The peanut oil mainly comprised α-tocopherol and γ-tocopherol, which accounted for 83.21–86.56% of the total content. The total tocopherol content decreased in the order of pressed oil > caproic acid demulsified oil > soxhlet-extracted oil. The total tocopherol content of the caproic acid demulsified oil was 3.23% lower than that of the pressed oil, which might have been due to the former process being conducted at a higher temperature, resulting in some tocopherols undergoing reactions to reduce their final content. Meanwhile, the total tocopherol content of the caproic acid demulsified oil was higher than that of the soxhlet-extracted oil. This was due to the tocopherol being dissolved in the peanut oil, and the POB, as a storage lipid, not being destroyed during the AEE process. The POB structure and composition were complete, which can avoid the interaction of tocopherol with polysaccharides and proteins, ultimately reducing the tocopherol loss [51,52,53].

### 3.9. Oxidation Stability

**Figure 9:**
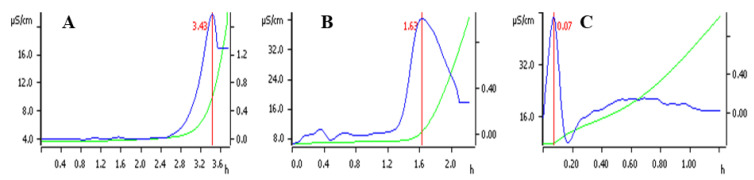
Accelerated oxidation diagram of crude peanut oil extracted by different methods ((**A**–**C**) represent pressed oil, soxhlet-extracted oil, and caproic acid demulsified oil, respectively). The red line, green curve and blue curve represent induction time, Rancimat curve and 2nd derivative curve respectively.

Accelerated oxidation experiments were conducted on the three types of peanut oil and the results are shown in Figure 9. Air was continuously injected into the oil at a high temperature (120 °C) and the volatile substances produced by the oil were introduced into pure water through the air. During this process, the conductivity of the pure water was continuously observed and the inflection point of the oxidation curve was detected, with the latter taken as the oxidation induction time, which reflects the oil stability [54]. The oxidation induction times of the pressed oil, soxhlet-extracted oil, and caproic acid demulsified oil were 3.43, 1.63, and 0.07 h, respectively. Therefore, the oxidation induction time of the caproic acid demulsified oil was significantly lower than that of the other two peanut oils. During the caproic acid demulsification process, a small amount of caproic acid entered the peanut oil, which induced the oil oxidation reaction during the oxidation induction, resulting in a significant decrease in the induction time. This caproic acid can be removed by subsequent oil refining, which can improve the oxidative stability of the demulsified oil.

## 4. Conclusions

Among even-carbon fatty acids with carbon chain lengths below ten, caproic acid afforded the highest demulsification rate of the POBs obtained via AEE (Viscozyme L). Through RSM optimization, the highest demulsification rate of 97.87% was obtained under optimal experimental conditions (caproic acid concentration, 0.22%; solid-to-liquid ratio, 1:4.7 (*w*/*v*); time, 61 min; and temperature, 79 °C). Caproic acid not only reduced the reaction system pH, but also caused the OBIPs to aggregate. Simultaneously, with an increasing caproic acid concentration, the interfacial tension and viscoelasticity of the interface film were significantly reduced, resulting in POB demulsification. During the POB demulsification, caproic acid addition, oil hydrolysis, and high temperatures led to increased acid and peroxide values for the caproic acid demulsified oil. Furthermore, the unsaturated fatty acid content and oxidation induction time were decreased, but the tocopherol and tocotrienol contents were effectively retained. The quality of the caproic acid demulsified oil could be improved by removing the free fatty acids through oil refining. Fatty acid demulsification provided a novel strategy and technology for the demulsification of POBs extracted via AEE.

## Figures and Tables

**Table 1:** Process variables and their levels used in Box–Behnken design.

Variables	Factor	Coded Levels		
		−1	0	1
X_1_	Concentration (%)	0.10	0.20	0.30
X_2_	Materials-to-liquid ratio (*w*/*v*)	1:4	1:5	1:6
X_3_	Time (min)	40	60	80
X_4_	Temperature (°C)	60	70	80

**Table 2:** Box–Behnken design matrix experiments and demulsification rate results.

Run	Variable				Response, Y
	X_1_	X_2_	X_3_	X_4_	
1	−1	0	0	−1	89.00 ± 0.21
2	−1	1	0	0	91.44 ± 0.18
3	−1	−1	0	0	92.67 ± 0.33
4	−1	0	1	0	92.51 ± 0.18
5	−1	0	0	1	93.89 ± 0.20
6	−1	0	−1	0	87.08 ± 0.13
7	0	−1	0	−1	91.23 ± 0.37
8	0	−1	1	0	94.56 ± 0.25
9	0	0	0	0	97.15 ± 0.43
10	0	0	0	0	97.00 ± 0.37
11	0	0	1	1	97.83 ± 0.55
12	0	0	0	0	96.81 ± 0.28
13	0	−1	0	1	96.11 ± 0.34
14	0	1	0	−1	92.18 ± 0.22
15	0	1	−1	0	93.00 ± 0.17
16	0	−1	−1	0	90.34 ± 0.31
17	0	0	−1	−1	89.58 ± 0.10
18	0	1	0	1	95.83 ± 0.50
19	0	1	1	0	94.66 ± 0.34
20	0	0	−1	1	96.12 ± 0.15
21	0	0	0	0	96.59 ± 0.26
22	0	0	0	0	95.99 ± 0.34
23	0	0	1	−1	93.89 ± 0.42
24	1	0	−1	0	92.58 ± 0.19
25	1	0	0	1	95.37 ± 0.29
26	1	0	1	0	95.42 ± 0.31
27	1	0	0	−1	92.21 ± 0.20
28	1	−1	0	0	93.60 ± 0.22
29	1	1	0	0	94.76 ± 0.26

**Table 3:** ANOVA for the RSM model. * and ** stand for *p* < 0.05 and *p* < 0.01, respectively.

Source	Sum of Squares	Degree of Freedom	Mean Squares	F Value	*p* Value	Significance
Model	191.71	14	13.69	18.78	<0.0001	**
X_1_	25.09	1	25.09	34.40	<0.0001	**
X_2_	0.94	1	0.94	1.29	0.2751	
X_3_	33.90	1	33.90	46.49	<0.0001	**
X_4_	61.02	1	61.02	83.49	<0.0001	**
X_1_X_2_	1.43	1	1.43	1.96	0.1835	
X_1_X_3_	1.68	1	1.68	2.30	0.1517	
X_1_X_4_	0.75	1	0.75	1.03	0.3283	
X_2_X_3_	1.64	1	1.64	2.25	0.1561	
X_2_X_4_	0.38	1	0.38	0.52	0.4833	
X_3_X_4_	1.69	1	1.69	2.32	0.1502	
X_1_^2^	47.24	1	47.24	64.77	<0.0001	**
X_2_^2^	13.97	1	13.97	19.15	0.0006	*
X_3_^2^	21.45	1	21.45	29.41	<0.0001	**
X_4_^2^	7.99	1	7.99	10.95	0.0052	**
Residual (error)	10.21	14	0.73			
Lack of fit	9.39	10	0.94	4.58	0.0779	No significance
Pure error	0.82	4	0.21			
Total	201.92	28				

**Table 4:** Physicochemical indexes of crude peanut oil extracted by different methods.

Index	Pressed Oil	Soxhlet-Extracted Oil	Caproic Acid Demulsified Oil
Acid value (mg KOH/g)	0.18 ± 0.03 ^b^	0.38 ± 0.02 ^b^	0.62 ± 0.06 ^a^
Peroxide value (g/100 g)	0.07 ± 0.00 ^b^	0.08 ± 0.00 ^b^	0.23 ± 0.00 ^a^

Different letters in the same row mean a significant difference (*p* < 0.05).

**Table 5:** Fatty acid composition of crude peanut oil extracted by different methods.

Fatty Acid	Pressed Oil	Soxhlet-Extracted Oil	Caproic Acid Demulsified Oil
Palmitic acid (C16:0)	11.89 ± 0.01 ^c^	12.43 ± 0.03 ^b^	12.69 ± 0.02 ^a^
Stearic acid (C18:0)	3.65 ± 0.01 ^c^	3.82 ± 0.02 ^b^	3.94 ± 0.01 ^a^
Oleic acid (C18:1)	38.04 ± 0.10 ^a^	37.75 ± 0.01 ^b^	38.19 ± 0.04 ^a^
Linoleic acid (C18:2)	40.67 ± 0.03 ^a^	40.25 ± 0.013 ^b^	38.90 ± 0.03 ^c^
Arachidic acid (C20:0)	1.47 ± 0.03 ^b^	1.44 ± 0.00 ^b^	1.49 ± 0.01 ^a^
Arachidonic acid (C20:1)	0.77 ± 0.01 ^a^	0.82 ± 0.02 ^a^	0.71 ± 0.12 ^a^
Behenic acid (C22:0)	2.29 ± 0.03 ^a^	2.28 ± 0.01 ^a^	2.33 ± 0.09 ^a^
Erucic acid (C22:1)	0.00 ± 0.00	0.00 ± 0.00	0.25 ± 0.02
Lignoceric acid (C24:0)	1.23 ± 0.04 ^b^	1.22 ± 0.04 ^b^	1.50 ± 0.03 ^a^
MUFA	38.81 ± 0.12 ^b^	38.57 ± 0.03 ^c^	39.16 ± 0.10 ^a^
PUFA	40.67 ± 0.03 ^a^	40.25 ± 0.01 ^b^	38.90 ± 0.03 ^c^
UFA	79.41 ± 0.09 ^a^	78.82 ± 0.05 ^b^	78.05 ± 0.07 ^c^
SFA	20.53 ± 0.09 ^c^	21.18 ± 0.05 ^b^	21.95 ± 0.07 ^a^
UFA/SFA	3.87 ± 0.02 ^a^	3.72 ± 0.01 ^b^	3.56 ± 0.01 ^c^
O/L	0.94 ± 0.00 ^b^	0.94 ± 0.68 ^b^	0.98 ± 0.00 ^a^

Different letters in the same row mean a significant difference (*p* < 0.05).

**Table 6:** Tocopherols content of crude peanut oil extracted by different methods.

	Pressed Oil	Soxhlet-Extracted Oil	Caproic Acid Demulsified Oil
α-tocopherol (µg/g)	5.61 ± 0.44 ^a^	5.37 ± 0.03 ^b^	5.62 ± 0.01 ^a^
α-tocotrienol (µg/g)	0.13 ± 0.01 ^a^	0.10 ± 0.01 ^b^	0.08 ± 0.00 ^b^
β-tocopherol (µg/g)	0.41 ± 0.00 ^a^	0.44 ± 0.02 ^a^	0.32 ± 0.00 ^b^
γ-tocopherol (µg/g)	4.04 ± 0.25 ^a^	3.45 ± 0.01 ^b^	3.98 ± 0.01 ^a^
γ-tocotrienol (µg/g)	0.81 ± 0.06 ^a^	0.78 ± 0.02 ^a^	0.78 ± 0.01 ^a^
δ-tocopherol (µg/g)	0.46 ± 0.00 ^a^	0.46 ± 0.00 ^a^	0.31 ± 0.00 ^b^
Total content (µg/g)	11.46 ± 0.08 ^a^	10.60 ± 0.05 ^c^	11.09 ± 0.02 ^b^

Different letters in the same row mean a significant difference (*p* < 0.05).

## Data Availability

All related data and methods are presented in this paper. Additional inquiries should be addressed to the corresponding author.
